# Sugar Transporter HmSWEET8 Cooperates with HmSTP1 to Enhance Powdery Mildew Susceptibility in *Heracleum moellendorffii* Hance

**DOI:** 10.3390/plants13162302

**Published:** 2024-08-19

**Authors:** Hanbing Liu, Junxia Liu, Xiaohui Si, Shuhong Zhang, Lili Zhang, Xuejiao Tong, Xihong Yu, Xinmei Jiang, Yao Cheng

**Affiliations:** 1 College of Horticulture and Landscape Architecture, Northeast Agricultural University, Harbin 150030, China; liuhanbing3291@163.com (H.L.); 15137337297@163.com (J.L.); ssixiaohui@163.com (X.S.); 15668319273@163.com (S.Z.); txjslzz1314@neau.edu.cn (X.T.); yxhong001@163.com (X.Y.); 2 Key Laboratory of Biology and Genetic Improvement of Horticulture Crops (Northeast Region), Ministry of Agriculture and Rural Afairs, Northeast Agricultural University, Harbin 150030, China; 3 College of Agriculture, Northeast Agricultural University, Harbin 150030, China; zhanglilizw@163.com

**Keywords:** *Heracleum moellendorffii* Hance, *Eeysiphe heraclei*, glucose, sugar transporter protein, susceptibility

## Abstract

The powdery mildew caused by *Eeysiphe heraclei* is a serious concern in *Heracleum moellendorffii* Hance. Therefore, exploring the mechanisms underlying sugar efflux from host cells to the fungus during the plant–fungus interaction showed great significance. The study successfully cloned *HmSWEET8* and *HmSTP1* genes based on RNA-seq technology. The complementation assays in yeast EBY.VW4000 found HmSWEET8 and HmSTP1 transporting hexose. Over-expressing or silencing *HmSWEET8* in *H. moellendorffii* leaves increased or decreased powdery mildew susceptibility by changing glucose concentration in infective sites. Meanwhile, over-expressing *HmSTP1* in *H. moellendorffii* leaves also increased powdery mildew susceptibility by elevating the glucose content of infective areas. Additionally, *HmSTP1* expression was up-regulated obviously in *HmSWEET8* over-expressed plants and inhibited significantly in *HmSWEET8* silenced plants. Co-expressing *HmSWEET8* and *HmSTP1* genes significantly increased powdery mildew susceptibility compared with over-expressed *HmSWEET8* or *HmSTP1* plants alone. The results demonstrated that HmSTP1 may assist with HmSWEET8 to promote *E. heraclei* infection. Consequently, the infection caused by *E. heraclei* resulted in the activation of *HmSWEET8*, leading to an increased transfer of glucose to the apoplasmic spaces at the sites of infection, then, HmSTP1 facilitated the transport of glucose into host cells, promoting powdery mildew infection.

## 1. Introduction

*Heracleum moellendorffii* Hance is classified under the order Apiaceae and is renowned not only for its palatable flavor but also for its abundance of flavonoids and coumarins, which exhibit significant anti-cancer properties [1]. The infestation of powdery mildew, induced by *Erysiphe heraclei*, poses a substantial threat to the developmental progression of *H. moellendorffii*, as it infiltrates diverse plant components such as seeds, flowers, and leaves, leading to compromised plant quality and yield reductions ranging from 10% to 40% [2], and restricting the large-scale planting. Currently, the ability of resistance (R) genes to resist pathogens is usually limited by the capacity of the pathogen to neutralize the plant’s immune response. Numerous researchers have thus examined and utilized the susceptibility genes (S-genes) that enhance the powdery mildew infection by increasing the pathogenic attack [3].

Microbial pathogens strategically exploit carbohydrates from the host to promote infection and proliferation in host cells [4,5]. Sugar transporters play a vital role in facilitating access to diverse sugar-dependent tissues and cells within the plant [6,7]. SWEETs (sugar will eventually be exported transporters) play a critical role as sugar transporters in plants, facilitating sugar transport and the regulation of key physiological processes throughout plant development, particularly in the context of plant–pathogen interactions [8]. The “pathogen starvation” and “sugar signaling” two concepts were proposed to explain sugar-mediated pathogen resistance. The “pathogen starvation” hypothesis indicates that fungal and bacterial pathogens can hijack sugars to promote infection and colonization by inducing *SWEET* over-expression, enhancing host susceptibility [5]. In many instances, SWEET sugar transporters are exploited by pathogens for the transport of sugars, without the transporter’s voluntary involvement [9]. For instance, bacterial blight in rice and cotton caused by *Xanthomonas* can utilize the TAL effectors to significantly induce *SWEET* gene expression, which then transports large amounts of sugars to extracellular spaces for pathogen proliferation [10,11,12,13]. In addition to bacteria, necrotizing fungi often induce *SWEET* gene upregulation accompanied by increased sugar accumulation in the fungal infection site and facilitate hyphal expansion and conidiophore germination [14]. Furthermore, the *SWEET* genes also participate in the “sugar signaling” pathway [15,16]. For instance, sugar accumulation in leaves of *Arabidopsis thaliana* sweet mutant activated salicylic-acid mediating defense response to inhibit fungal hemibiotroph *Colletotrichum higginsianum* (*Ch*) infection [17]. These findings therefore demonstrate that *SWEETs* may mediate different interaction mechanisms between pathogens and plants.

Powdery mildew and stipe rust are examples of fungal biotrophs that produce a haustorium structure to absorb sugars from the plant–haustorium interface [18,19]. Sugar must be transported from the host cytoplasm across the extra haustorial membrane (EHM), that is the host–pathogen interface, into the EHMx before it can be taken by haustoria [20]. Studies have shown that the sugar transporter protein (STP) is capable of transferring sugars across the EHMx from host cells to biotrophic fungus [21,22,23]. For example, in Arabidopsis, ER-localized sugar transporter AtSTP8 can be recruited to the extra haustorial membrane (EHM) where it may be involved in sugar acquisition by haustoria of powdery mildew [20]. The infective regions of powdery mildew are recognized as sink organs, requiring ample sugar for their expansion [24]. Therefore, the pathogen has to manipulate another sugar transporter which can mediate long-distance transport of sugar from the source into the infective areas [25]. In the process, SWEET transporters can facilitate sugar transport from high to low concentration over long distances [26], assuming that it may be recruited to supply sugars for powdery mildew [27]. *Arbuscular mycorrhizal* (AM) fungi utilize MtSWEET1b to transport glucose into the peri-arbuscular spaces, where it can be taken up by the AM fungus via monosaccharide transporter-like MST2. These fungi develop haustoria in host cells, exhibiting a structural arrangement similar to powdery mildew [28].

At present, several *SWEET* genes are induced dramatically by biotrophic fungi [27], however, the underlying molecular mechanisms are not clear. Therefore, we hypothesize that powdery mildew and AM fungus may share a molecular mechanism by which the former may control *SWEET* transporters to transfer sugars into the powdery mildew’s infectious site and collaborate with another sugar transporter to facilitate infection. In the current study, according to previously reported RNA-seq data in *H. moellendorffii* following *E. heraclei* infection [1], we cloned *HmSWEET8* and *HmSTP1* genes successfully in *H. moellendorffii* and investigated the sugar-transporting functions of HmSWEET8 and HmSTP1 in yeast. Additionally, the results of transient expression and silencing assays showed that HmSTP1 may assist with HmSWEET8 to promote *E. heraclei* infection by increasing the glucose content of infective sites. These findings provide new insights into the process of nutritional competition between *E. heraclei* and its host, *H. moellendorffii*, and provide innovative strategies for managing the infection.

## 2. Results

### 2.1. Cloning of HmSWEET8 Gene in H. moellendorffii

**Figure 1:**
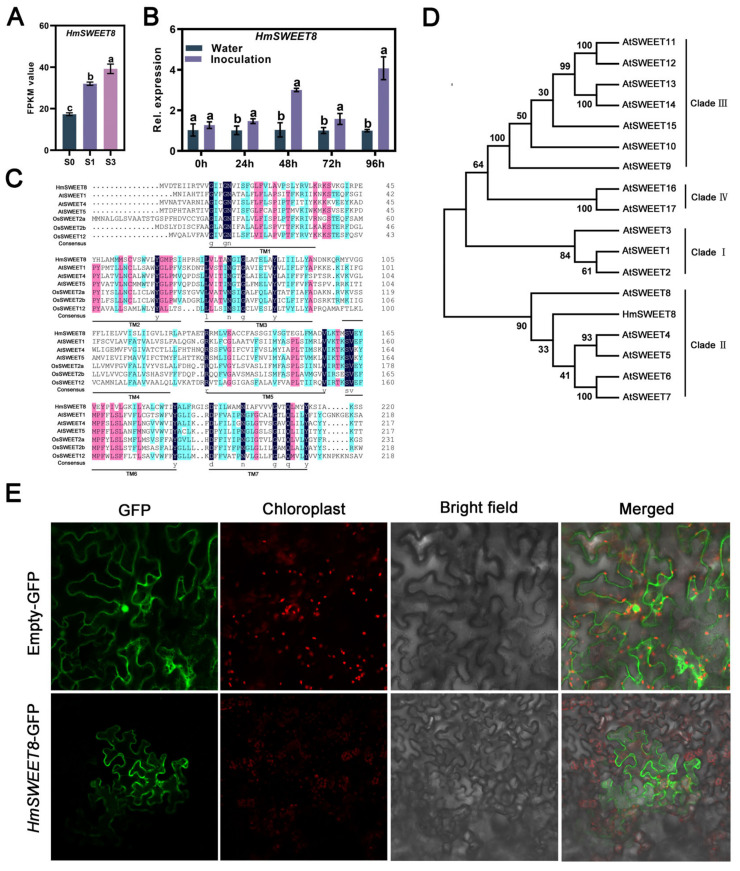
Cloning of *HmSWEET8* gene in *Heracleum moellendorffii* Hance: (**A**) The FPKM value of *HmSWEET8* in S0, S1, and S3 infected leaves. Leaves infected with *Eeysiphe heraclei* exhibiting varying degrees of disease. S1: The area of occurring colonies accounted for less than 10% of the whole leaf area; S3: the area of occurring colonies accounts for 30–50% of the whole leaf area; S0: the leaves with the water treatment were collected as the control. Statistical significance was indicated by different letters, as determined by one-way ANOVA measured at *p* < 0.05. (**B**) The expression level of *HmSWEET8* after *E. heraclei* infection. Statistical significance was indicated by different letters, as determined by Student’s *t*-test measured at *p* < 0.05. (**C**) Multiple sequences alignment of HmSWEET8 with *A. thaliana* and *Oryza sativa* Japonica Group. SWEET protein sequences. HmSWEET8 (accession number: OM515217) was obtained from *H. moellendorffii*. AtSWEET1, AtSWEET4, and AtSWEET5 (accession number: Q8L9J7, Q944M5, and Q9FM10, respectively), were obtained from *A. thaliana*. OsSWEET2a, OsSWEET2b, and OsSWEET12 (accession number: Q5JJY5, Q5N8J1, and Q10LI8, respectively), were obtained from *Oryza sativa* Japonica Group. (**D**) The phylogenetic tree was constructed using HmSWEET8 and *A. thaliana* SWEET protein sequences. (**E**) Subcellular localization of HmSWEET8 in *Nicotiana benthamiana* epidermal cells. Scale bar: 20 μm.

Based on the previously published RNA-seq data in *H. moellendorffii* following *E. heraclei* infection, a SWEET sugar transporter HmSWEET8 was induced dramatically after *E. heraclei* infection (Figure 1A), and artificial inoculation of *E. heraclei* could induce the expression of *HmSWEET8* obviously (Figure 1B), indicating that *HmSWEET8* gene may involve in the interaction of *H. moellendorffii*–*E. heraclei*. Therefore, we cloned the *HmSWEET8* gene successfully by designing a specific primer. The HmSWEET8 protein encoded seven complete transmembrane structures (Figure 1C), and phylogenetic analysis showed that HmSWEET8 was grouped with SWEET proteins obtained from A. thaliana in Clade II (Figure 1D). Subcellular location found HmSWEET8 encoding a plasma membrane protein (Figure 1E).

### 2.2. HmSWEET8 Could Transport Hexose

**Figure 2:**
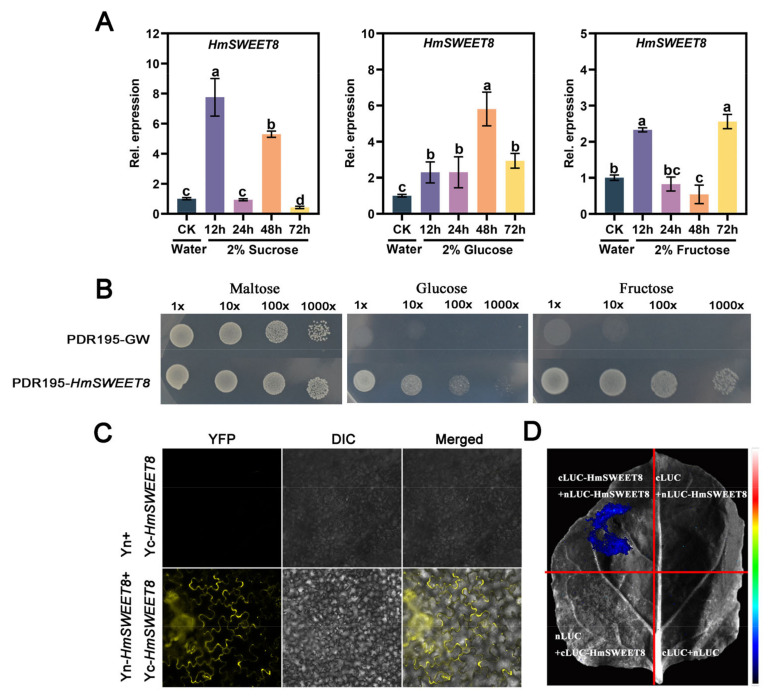
The confirmation of sugar transporting ability mediated by HmSWEET8: (**A**) The expression of *HmSWEE8* with different 2% sugar treatments. Statistical significance was indicated by different letters, as determined by one-way ANOVA measured at *p* < 0.05. (**B**) The complementation assays in the hexose transport-deficient yeast strain EBY.VW4000. (**C**) Bimolecular fluorescence complementation (BiFC) was used for detection of HmSWEET8 homodimer. Scale bar: 20 μm. (**D**) Luciferase Complementation Assay (LCA) was used for detection of HmSWEET8 homodimer.

The sugar-transporting ability of HmSWEET8 was explored in the next experiment. The 2% sucrose, fructose, and glucose treatments could induce *HmSWEET8* expression obviously compared with water treatment (Figure 2A). The complementation assays in the hexose transport-deficient yeast strain EBY.VW4000 found that pDR195-*HmSWEET8* expression restored EBY.VW4000 growth on 2% glucose and fructose (Figure 2B), suggesting that HmSWEET8 could transport glucose and fructose. Bimolecular fluorescence complementation (BiFC) found that the YFP signal was observed in *Nicotiana benthamiana* epidermal cells when co-expressed Yn-*HmSWEET8* and Yc-*HmSWEET8* (Figure 2C). Luciferase Complementation Assay (LCA) experiments found that LCA activity was observed when co-expressed nLUC-*HmSWEET8* and cLUC-*HmSWEET8* in *N. benthamiana* leaves (Figure 2D). These results demonstrated that HmSWEET8 could form the homodimer to perform transport functions.

### 2.3. HmSWEET8 Promoted E. heraclei Infection in H. moellendorffii

**Figure 3:**
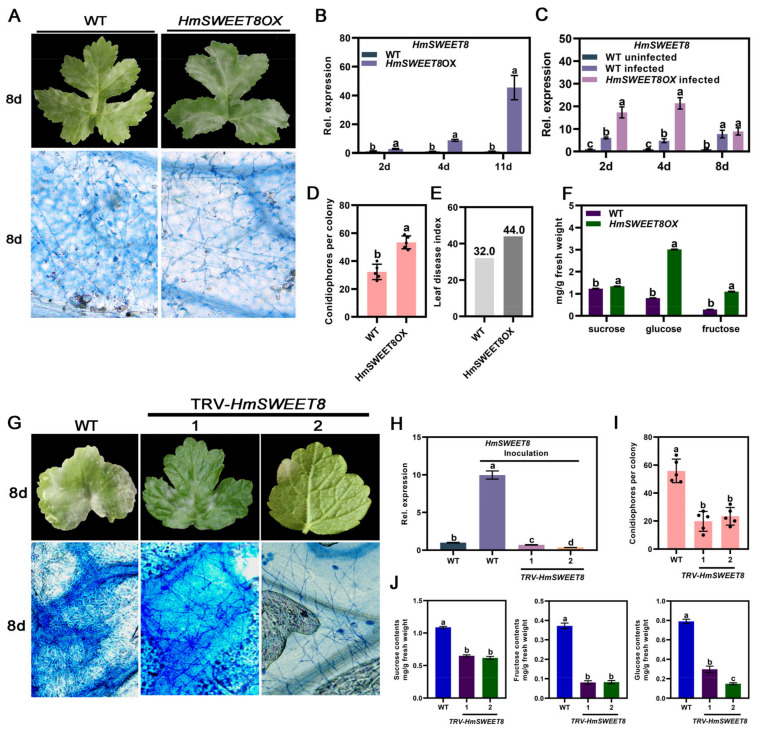
*HmSWEET8* gene was transiently over-expressed in *H. moellendorffii* leaves: (**A**) The typical genotypes of *H. moellendorffii* leaves were observed after *E. heraclei* infection at 8 d. Scale bar: 400 μm. (**B**) The expression of *HmSWEET8* without *E. heraclei* infection. Statistical significance was indicated by different letters, as determined by Student’s *t*-test measured at *p* < 0.05. (**C**) The expression of *HmSWEET8* after artificial inoculation *E. heraclei.* Statistical significance was indicated by different letters, as determined by one-way ANOVA measured at *p* < 0.05. (**D**,**E**) The calculation of conidiophores per colony (**D**) and leaf disease index (**E**). Statistical significance was indicated by different letters, as determined by Student’s *t*-test measured at *p* < 0.05. (**F**) The soluble sugar content of infected leaves was measured. Statistical significance was indicated by different letters, as determined by Student’s *t*-test measured at *p* < 0.05. (**G**) The typical genotypes of *H. moellendorffii* leaves were observed in *HmSWET8* (1**#**, 2**#**) silenced seedlings after *E. heraclei* infection. Scale bar: 400 μm. (**H**) The expression of *HmSWEET8* was measured. Statistical significance was indicated by different letters, as determined by one-way ANOVA measured at *p* < 0.05. (**I**) The calculation of conidiophores per colony in infected leaves. Statistical significance was indicated by different letters, as determined by one-way ANOVA measured at *p* < 0.05. (**J**) The soluble sugar content of infected leaves was measured. Statistical significance was indicated by different letters, as determined by one-way ANOVA measured at *p* < 0.05.

To verify the performed function mediated by HmSWEET8 in the interaction of *H. moellendorffii*–*E. heraclei*, the *HmSWEET8* gene was transiently over-expressing in *H. moellendorffii* leaves. *HmSWEET8* expression was elevated significantly in the over-expression of plants (*HmSWEET8*OX) compared with WT plants (Figure 3B). After the artificial inoculation of *E. heraclei*, *HmSWEET8* expression in *HmSWEET8*OX plants was induced dramatically (Figure 3C). The powdery mildew occurred seriously in *HmSWEET8*OX plants, and higher leaf disease index and conidiophores per colony were observed compared with WT plants at 8d (Figure 3A,D,E). These results demonstrated that *HmSWEET8* was a susceptible gene to promote *E. heraclei* infection. The soluble sugar content of *H. moellendorffii* infected leaves was measured. Compared with WT plants, the contents of soluble sugar, especially glucose, were obviously accumulated in *HmSWEET8*OX plants (Figure 3F). The TRV silenced system was used to silence *HmSWEET8* expression in *H. moellendorffii* leaves. *HmSWEET8* expression in TRV-*HmSWEET8* plants was inhibited significantly, and a decreased number of mycelia and conidiophores was observed compared with WT plants (Figure 3G–I). The sugar concentration, especially hexose, of infected leaves decreased significantly in TRV-*HmSWEET8* plants (Figure 3J). These results indicated that *HmSWEET8* over-expression contributed to *E. heraclei* infection by elevating the glucose content in infective sites.

### 2.4. HmSTP1 Promoted E. heraclei Infection

**Figure 4:**
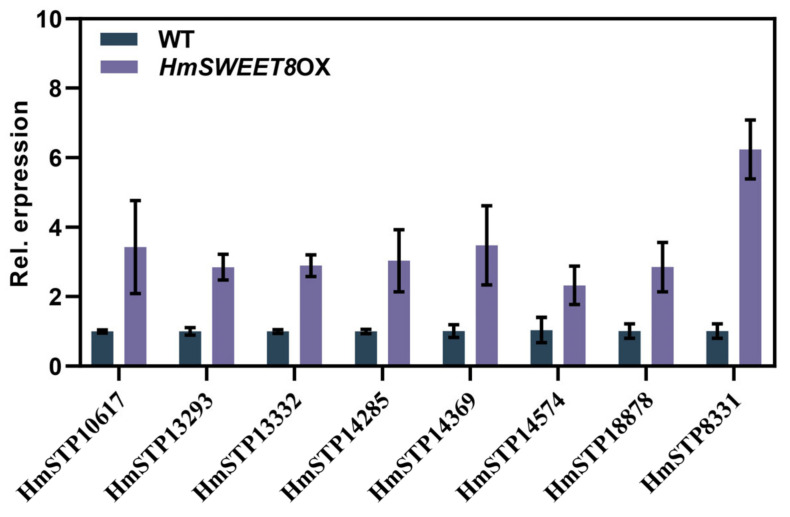
*HmSTP* genes were all induced significantly in *HmSWEET8OX* plants.

Prior research has shown that powdery mildew exploits STP sugar transporters to facilitate the movement of sugars from host cells into the powdery mildew across the EHMx. Therefore, we assumed that HmSTPs may be involved in the powdery mildew susceptibility mediated by HmSWEET8 protein in *H. moellendorffii*. Interestingly, eight *HmSTP* genes were all induced in *HmSWEET8OX* plants compared with WT plants (Figure 4). The results indicated that HmSWEET8 and HmSTPs may have synergistic effects in transporting sugars in *H. moellendorffii*.

**Figure 5:**
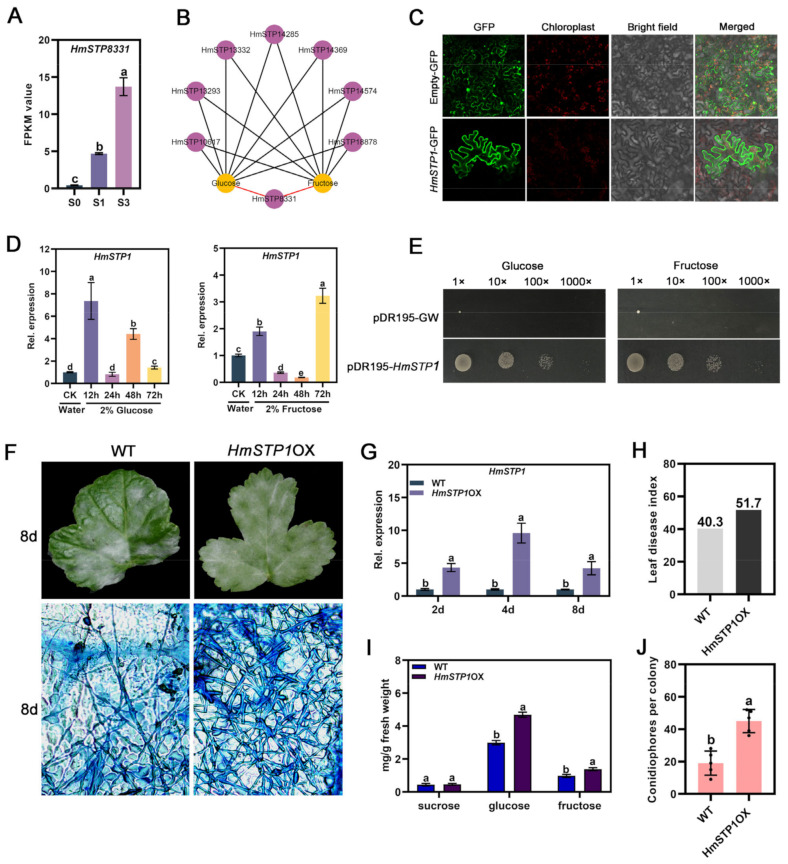
HmSTP1 protein responding to *E. heraclei* infection in *H. moellendorffii*: (**A**) The FPKM value of *HmSTP8331* in S0, S1, and S3 infected leaves. Statistical significance was indicated by different letters, as determined by one-way ANOVA measured at *p* < 0.05. (**B**) The correlation network analysis between *HmSTP8331* expression with hexose contents, the red line: r^2^ > 0.90. (**C**) Subcellular localization of HmSTP1 in *N. benthamiana* epidermal cells. Scale bar: 20 μm. (**D**) The expression of *HmSTP1* with different 2% sugar treatments. Statistical significance was indicated by different letters, as determined by one-way ANOVA measured at *p* < 0.05. (**E**) Complementation of yeast EBY.VW4000 with HmSTP1. (**F**) The typical genotypes of *H. moellendorffii* leaves were observed after *E. heraclei* infection at 8 d. Scale bar: 50 μm. (**G**) The expression of *HmSTP1* after artificial inoculation *E. heraclei*. Statistical significance was indicated by different letters, as determined by Student’s *t*-test measured at *p* < 0.05. (**H**) The calculation of leaf disease index in infected leaves. (**I**) The soluble sugar content of infected leaves was measured. Statistical significance was indicated by different letters, as determined by Student’s *t*-test measured at *p* < 0.05. (**J**) The calculation of conidiophores per colony in infected leaves. Statistical significance was indicated by different letters, as determined by Student’s *t*-test measured at *p* < 0.05.

HmSTP proteins were identified based on the RNA-seq analysis (Table S1). Among them, *HmSTP8331* expression was induced dramatically in S1 and S3 infected leaves, and positively correlated with the accumulation of hexose (Figure 5A,B, Table S2). Therefore, *HmSTP1* (*HmSTP8331*) was selected as the target gene for further research. Subcellular location experiment found HmSTP1 encoding a plasma membrane protein (Figure 5C). *HmSTP1* expression could be induced by 2% glucose and fructose treatments (Figure 5D), and the complementation assays in the yeast strain EBY.VW4000 found that HmSTP1 could transport glucose and fructose (Figure 5E). With over-expressing *HmSTP1* in *H. moellendorffii* leaves, the powdery mildew severity increased in *HmSTP1*OX plants with more visible mycelia and conidiophores at 8 d, compared to WT (Figure 5F–H,J). Furthermore, the hexose level, especially glucose, of infected leaves significantly increased in *HmSTP1*OX plants (Figure 5I), suggesting that *HmSTP1* as the susceptible gene promoted *E. heraclei* infection by elevating the glucose content in infective sites.

### 2.5. HmSTP1 May Assist with HmSWEET8 to Promote E. heraclei Infection

**Figure 6:**
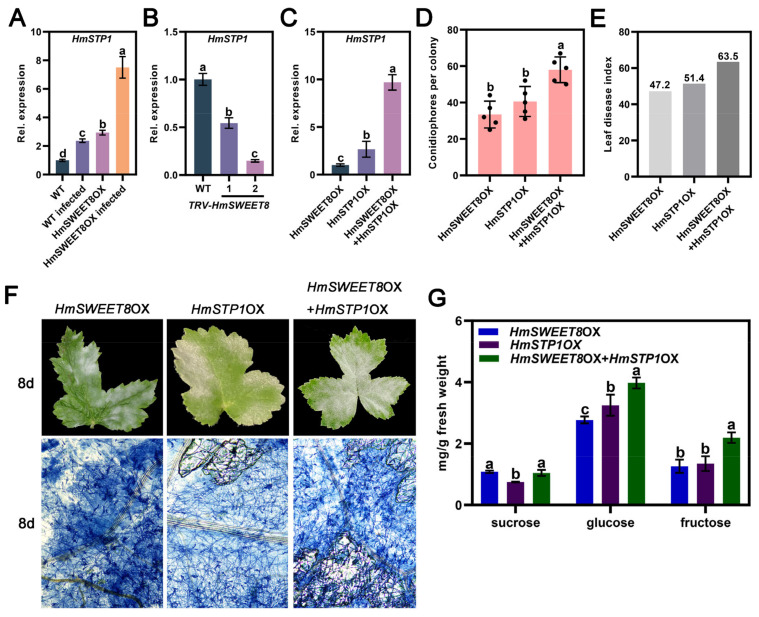
*HmSWEET8* and *HmSTP1* genes were transiently co-expressed in *H. moellendorffii* leaves: (**A**) The expression of *HmSTP1* in *HmSWEET8* over-expressed plants. Statistical significance was indicated by different letters, as determined by one-way ANOVA measured at *p* < 0.05. (**B**) The expression of *HmSTP1* in *HmSWEET8* silenced plants. Statistical significance was indicated by different letters, as determined by one-way ANOVA measured at *p* < 0.05. (**C**) The expression of *HmSTP1* in co-expressed *HmSWEET8* and *HmSTP1* plants. Statistical significance was indicated by different letters, as determined by one-way ANOVA measured at *p* < 0.05. (**D**,**E**) The calculation of conidiophores per colony and leaf disease index (**E**) in infected leaves. Statistical significance was indicated by different letters, as determined by one-way ANOVA measured at *p* < 0.05. (**F**) The typical genotypes of *H. moellendorffii* leaves were observed after *E. heraclei* infection at 8 d. Scale bar: 400 μm. (**G**) The soluble sugar content of infected leaves was measured. Statistical significance was indicated by different letters, as determined by one-way ANOVA measured at *p* < 0.05.

Further, we explored the interaction between HmSWEET8 and HmSTP1 in powdery mildew infection. The transiently over-expressing *HmSWEET8* gene in *H. moellendorffii* induced *HmSTP1* up-regulation and gene expression was significantly enhanced by powdery mildew infection (Figure 6A). *HmSWEET8* gene silencing in *H. moellendorffii* suppressed *HmTP1* expression (Figure 6B). Consequently, there may exist synergistic effects between HmSWEET8 and HmSTP1 in transporting sugars into the infective site of powdery mildew. Co-expressing *HmSWEET8* and *HmSTP1* genes elevated *HmSTP1* expression dramatically compared with over-expressing *HmSWEET8* or *HmSTP1* alone (Figure 6C). The powdery mildew severity increased in co-expressed *HmSWEET8OX* and *HmSTP1*OX plants with more visible conidiophores and higher leaf disease index at 8 d, compared to *HmSWEET8*OX or *HmSTP1*OX plants (Figure 6D–F). Furthermore, the hexose level, especially glucose, of infected leaves significantly increased in co-expressed *HmSWEET8*OX and *HmSTP1*OX plants (Figure 6G). Consequently, we thought that HmSWEET8 and HmSTP1 may have synergistic effects in transporting sugars to promote powdery mildew infection. After the *E. heraclei* infection, the HmSWEET8 protein transported more glucose into apoplasmic spaces of infective areas, and the glucose was then transported to the interface of infection areas for the powdery mildew infection via HmSTP1 protein.

## 3. Discussion

Powdery mildew is an example of a fungal biotroph that forms a haustorium structure to absorb sugars from the plant–haustorium interface [20]. Sugar transporters play a crucial role in facilitating this process by enabling the transfer of sugars to different sugar-dependent tissues and cells [7]. In the study, the *HmSWEET8* gene significantly responded to *E. heraclei* infection, indicating that *HmSWEET8* may play important roles in the *E. heraclei* and *H. moellendorffii* interaction (Figure 1A,B). SWEET proteins are present at both the source and sink terminals of the phloem pathway, facilitating the transport of sugars from the phloem parenchyma cells to the apoplasmic spaces [26,29]. In the study, the over-expression of *HmSWEET8* promoted *E. heraclei* infection, and the glucose of infected leaves significantly increased in *HmSWEET8*OX plants (Figure 3A–F), and silenced mutants showed opposite results (Figure 3G–J). The findings indicated that the gene *HmSWEET8* was susceptible to powdery mildew infection and may carry more glucose into the infectious region [30]. Similar to AM fungus, *E. heraclei* infection induced *HmSWEET8* gene expression and may accelerate more glucose efflux into the apoplasmic spaces of infective areas of powdery mildew [28,31]. The study demonstrated that certain SWEET family members in *H. moellendorffii* may be recruited in the phloem loading process by *E. heraclei*, and *E. heraclei* retools a critical physiological function to gain sugars from the host at the site of infection.

The sugar efflux rate mediated by SWEET proteins may be enhanced by hexose transporters’ withdrawal from the host apoplasm [5]. In wheat and *Arabidopsis* leaves, the glucose uptake could be correlated with a change in hexose transporter gene expression [32,33]. In *Medicago truncatula*, MtSWEET1b exported glucose from the plant cortical cells into the peri-arbuscular space, where it can be taken up by the AM fungus via monosaccharide transporters like MST2 [34]. Over-expressing the *HmSWEET8* gene promoted *HmSTP* gene expression dramatically (Figure 4), and *HmSTP1* expression was significantly enhanced by powdery mildew infection (Figure 6A). Therefore, similar to previously reported research, there may exist synergistic effects between HmSWEET8 and HmSTP1 in transporting sugars into the site of powdery mildew infection. HmSTP1 was identified as the plasma membrane protein that transported the hexose (Figure 5C–E). Over-expression of *HmSTP1* elevated the hexose contents of infective sites, promoting powdery mildew infection (Figure 5F–J), which was consistent with previous studies [20,35]. Co-expressing *HmSWEET8* and *HmSTP1* genes increased the powdery mildew severity, and the glucose content was elevated significantly. The results seemingly indicated that HmSTP1 may assist with HmSWEET8 to promote *E. heraclei* infection. In the process, HmSWEET8 transported more glucose from adjacent cells into the apoplasmic spaces, and HmSTP1 may import the glucose into the haustorium or import the glucose into the cell that feeds the haustorium [5].

This result was not consistent with the “sugar starvation” hypothesis. In which, the microbial pathogen controls the secretion of hexose by SWEETs into the area between cell walls, where the microorganism utilizes hexoses as a source of nourishment and for reproduction. The STPs prevent the build-up of hexose in the cell wall by using secondary active transport to remove them [5]. The variability in outcomes may be attributed to how infections obtain nutrients [36]. It is crucial to recognize that the process of obtaining nutrients by symbiotic bacteria and necrotrophic and biotrophic pathogens may vary significantly [5]. Bacterial pathogens and necrotrophic fungi efficiently manipulate SWEET proteins during the pre-necrotic stage to transport a higher amount of sugars into the apoplasmic regions, providing nourishment for the pathogens [11,37]. STPs take up hexoses from apoplasmic space, depriving the fungus by changing sugar fluxes toward host cells and enhancing resistance to *Botrytis cinerea* [38], supporting the “sugar starvation” hypothesis. Different from bacterial pathogens, the powdery mildew relies on haustorial invasion turns the invaded host cell into a net sink for photoassimilates, and STPs absorb sugars from the apoplasmic space into the EHMs for infection [18,20,39], and the process needs SWEETs to generate a larger apoplasmic hexose pool for retrieval by STPs supporting the infection process [28,31]. Therefore, in the study, the sugar accumulation in the apoplasmic space, facilitated by HmSWEETs, established a consistent supply of hexose for the retrieval of STP into the cells during *E. heraclei* infection. Thus, it is possible that the sugars carried by HmSWEETs did not directly contribute to *E. heraclei* infection and instead relied on the aid of HmSTP.

## 4. Materials and Methods

### 4.1. Plant Material

*H. moellendorffii* susceptible plants were selected from the germplasm garden located in Haerbin City, China. The susceptible plants were cultivated (planting date was 8 May 2022) in the greenhouse of the Gardening Test Station with a 16/8 h (light/dark) photoperiod and a temperature range of 25/18 °C (day/night). The powdery mildew isolate, identified as *E. heraclei* [2], was separated and cultivated from infected *H. moellendorffii* leaves.

### 4.2. Isolation of HmSWEET8 and HmSTP1

The 783 bp and 1572 bp cDNA sequences of *HmSWEET8* and *HmSTP1* were amplified by primers, respectively. The design of primers was based on the sequence template of *H. moellendorffii* using Single Molecule Real-Time (SMRT) (BioProject accession: PRJNA1032719). The cDNA fragments were inserted into PMD19-T vector and sequenced (Takara, Shanghai, China).

### 4.3. Subcellular Localization

The pCAMBIA1300-*HmSWEET8*-sGFP and pCAMBIA1300-*HmSTP1*-sGFP vectors were constructed and transiently expressed in *Nicotiana benthamiana* via *Agrobacterium*-mediated transformation using the GV3101 strain (Angyu, Shanghai, China) as previously described [40]. The resultant GFP fusion proteins were then visualized using a laser-scanning confocal microscope (FV3000, Olympus, Tokyo, Japan).

### 4.4. Complementation Assays in Yeast

The PDR195-*HmSWEET8* and PDR195-*HmSTP1* recombinant plasmids were constructed and transformed into the hexose transport-deficient yeast strain EBY.VW4000. After the transformation, yeast cells underwent cultivation on SC-ura solid medium supplemented with 2% maltose at 30 °C for a duration of 3 days. Subsequently, for complementation growth assays, the cells were grown overnight in SC-ura liquid medium with a 2% maltose supplement, reaching an optical density of 1.0 at 600 nm (OD600). One milliliter of cells was suspended and adjusted with sterile water. Subsequently, serial dilutions (1×, 10×, 100×, and 1000×) were plated on SC-ura solid medium containing either 2% maltose, 2% glucose, and 2% fructose. After incubating for 3 days at 30 °C, the colony growth on the plates was evaluated.

### 4.5. Bimolecular Fluorescence Complementation (BiFC) Assay

The pCAMBIA1300-*HmSWEET8*-YFPN and pCAMBIA1300-*HmSWEET8*-YFPC vectors were constructed and co-expressed in *N. benthamiana* leaves using the GV3101 Agrobacterium strain. After 2–3 days of cultivation, the YFP signals were then visualized using a laser-scanning confocal microscope (FV3000, Olympus, Tokyo, Japan).

### 4.6. Luciferase Complementation Assay (LCA)

The pCAMBIA1300-*HmSWEET8*-nLUC and pCAMBIA1300-*HmSWEET8*-cLUC vectors were constructed and co-expressed in *N. benthamiana* leaves with the GV3101 Agrobacterium strain. Following a cultivation period of 2–3 days, the outcomes of LUA studies were captured utilizing an automated chemiluminescence image analysis system (Tanon 5200, Shanghai, China).

### 4.7. Transient Transformation of H. moellendorffii

The recombinant plasmids pCAMBIA1300-*HmSWEET8*-sGFP, pCAMBIA1300-*HmSTP1*-sGFP were transformed using the GV3101 *Agrobacterium* strain as previously reported in *H. moellendorffii* [1]. The cells were initially suspended at OD600 0.8 using MMA, followed by a 3 h dark incubation, before being transformed into *H. moellendorffii* leaves via the vacuum infiltration (SHZ-D, Shanghai, China). Subsequently, the *H. moellendorffii* leaves were cultured for 2 days at 22 °C in a light incubator (MGC-350HP-2, Shanghai, China), after which they were inoculated with *E. heraclei* spores at a concentration of 1 × 10^6^. Post-inoculation, samples were collected for gene expression analysis and quantification of soluble sugar content. Finally, a visual assessment of the disease condition caused by *E. heraclei* was performed.

### 4.8. Virus-Induced Gene Silencing (VIGS) in H. moellendorffii

A 300 bp fragment of *HmSWEET8* was ligated into TRV2 vectors. The empty TRV1 vector and recombinant plasmids were transformed into the GV3101 *Agrobacterium* strain. The cells were collected and suspended at an optical density of 600 nm (OD600) of 0.8 using MMA. Leaves of *H. moellendorffii* were excised, retaining only the roots with bud apices [41]. Subsequently, the suspended cell mixture was introduced into *H. moellendorffii* roots containing buds via vacuum infiltration (SHZ-D, Shanghai, China) [42]. Incisions were made in the roots using a knife before the vacuum step to facilitate the entry of suspended cells. During vacuum infiltration, the roots were immersed in the cell suspension, subjected to a 0.1 MPa vacuum for 10 min, and then swiftly depressurized to promote rapid inoculum penetration. This process was repeated once. The transformed roots were planted in a soil matrix and cultivated at 22 °C for 3 days under the dark condition and then shifted to normal conditions at 22 °C with the 16/10 h light/dark cycle. After a 15-day period, the emerging leaves were inoculated with a suspension of *E. heracleid* spores at a concentration of 1 × 10^6^, grown in a light incubator, and cultivated at a photoperiod of 16/8 h (light/dark) and a temperature of 25/18 °C (day/night) in the light incubator (MGC-350HP-2, Shanghai, China). Samples were collected for gene expression and soluble sugar analysis. Subsequently, observations were made to assess the disease status induced by *E. heraclei*.

### 4.9. E. heraclei Infection in H. moellendorffii

The disease index calculation, trypan blue staining observing the powdery mildew infection, and conidiophores per colony calculation were conducted as previously reported in *H. moellendorffii* [1].

### 4.10. Soluble Sugar Content Measurement in H. moellendorffii

The soluble sugar of fresh leaves in *H. moellendorffii* was extracted and measured contents as previously described [30]. The reference sugars were purchased from the HerbSubstance company (HerbSubstance, Chengdu, China).

### 4.11. Quantitative Real-Time PCR Analysis

The EasyPure Plant RNA Kit (TransGen Biotech, Beijing, China) was used to extract Plant RNA. The EasyScript One-Step gDNA Removal and cDNA Synthesis SuperMix (TransGen Biotech, Beijing, China) was used to synthesize cDNA Synthesis SuperMix. The selection of reference gene HmActin, qRT-PCR assays, and the calculation of relative gene expression were performed as previously described in *H. moellendorffii* [1].

### 4.12. Statistical Analysis

The Excel 2010 software was used to perform data statistics, and the GraphPad Prism 8.0.1 software was used to plot bar graphs. Differentiating letters were used to denote statistical significance with Student’s *t*-test or one-way analysis of variance (ANOVA) assessed at a significance level of *p* < 0.05. All primers used in this study are presented in Table S3.

## 5. Conclusions

**Figure 7:**
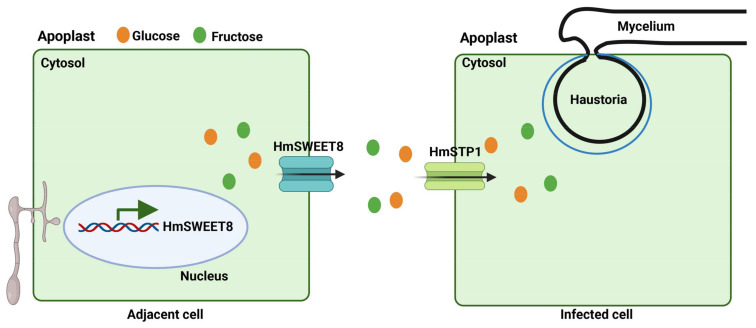
A working model portraying the underlying mechanisms by which *E. heraclei* manipulates host sugar transporters to acquire nutrients and promote fungal infection. *E. heraclei* infection activates the expression of *HmSWEET8 and HmSTP1*, then, *HmSWEEμmT8* protein transports more glucose into apoplasmic spaces of infective areas. HmSWEET8 generates a larger apoplasmic hexose pool and promotes HmSTP1 expression. The glucose then is transported into host cells via *HmSTP1* sugar transporter for promoting *E. heraclei* infection.

The objective of the research was to clarify the molecular processes by which sugar transporters and sugars react to the powdery mildew infection. qRT-PCR analysis found that *HmSWEET8* and *HmSTP1* were involved in *E. heraclei* infection. Complementation of yeast EBY.VW4000 and subcellular localization experiments demonstrated that HmSWEET8 and HmSTP1 localized the plasma membrane with hexose transporting activity. *HmSWEET8* and *HmSTP1* were determined to be susceptible genes by transporting more hexose to the infection areas of *E. heraclei*. After *E. heraclei* infection, HmSWEET8 transported more glucose into apoplasmic spaces of infective areas, and then transported to the interface of the infection areas for the powdery mildew infection via HmSTP1 protein. In the process, the glucose efflux mediated by HmSWEET8 may be enhanced by HmSTP1 (Figure 7).

## Data Availability

The data presented in this study are available upon request from the corresponding author.

## Supplementary Materials

The following supporting information can be downloaded at: https://www.mdpi.com/article/10.3390/plants13162302/s1, Table S1: STP sugar transporters were identified using RNA-seq analysis; Table S2: The Pearson’s correlation analysis between sugar transporters and soluble sugars; Table S3: Primer design of qRT-PCR and vector construction.
